# Efficacy of fluvastatin and aspirin for prevention of hormonally insensitive breast cancer

**DOI:** 10.1007/s10549-021-06229-0

**Published:** 2021-04-24

**Authors:** Anjana Bhardwaj, Matthew D. Embury, Raniv D. Rojo, Constance Albarracin, Isabelle Bedrosian

**Affiliations:** 1 Department of Breast Surgical Oncology, The University of Texas MD Anderson Cancer Center, Houston, TX USA; 2 College of Medicine, University of the Philippines Manila, Manila, Philippines; 3 Department of Pathology, The University of Texas MD Anderson Cancer Center, Houston, TX USA

**Keywords:** TNBC, Fluvastatin, Aspirin, SV40 C3TAg mice, Prevention

## Abstract

**Purpose:**

Primary prevention of hormonally insensitive breast cancers remains an important clinical need and repurposing existing low-toxicity drugs represents a low-cost, efficient strategy for meeting this goal. This study targeted the cholesterol pathway using fluvastatin, a cholesterol-lowering drug, and aspirin, an AMPK activator that acts as a brake in the cholesterol pathway, in a transgenic mouse model of triple-negative breast cancer (TNBC).

**Methods:**

Using SV40C3 TAg mice, the efficacy and mechanism of fluvastatin, aspirin, or both in combination were compared with vehicle alone.

**Results:**

Sixteen-weeks of fluvastatin treatment resulted in significant delay in onset of tumors (20 weeks vs. 16.8 weeks in vehicle treatment, *p* = 0.01) and inhibited tumor incidence and tumor multiplicity by 50% relative to the vehicle control. In animals that developed tumors, fluvastatin treatment inhibited tumor weight by 75% relative to vehicle control. Aspirin alone did not significantly affect tumor latency, tumor incidence or tumor burden compared to vehicle control. Fluvastatin and aspirin in combination delayed the onset of tumors but failed to inhibit tumor incidence and tumor multiplicity. The growth-inhibitory effects of fluvastatin were mediated through increased FAS/FASL mediated apoptotic cell death that was characterized by increased cleaved PARP and driven in part by depletion of an isoprenoid, geranyl geranyl pyrophosphate (GGPP).

**Conclusions:**

In line with NCI’s emphasis to repurpose low-toxicity drugs for prevention of cancer, fluvastatin was effective for prevention of TNBC and warrants further clinical testing. Aspirin did not provide chemopreventive benefit.

**Supplementary Information:**

The online version contains supplementary material available at 10.1007/s10549-021-06229-0.

## Introduction

Statins, drugs that target the cholesterol biosynthesis pathway, have become among the most widespread drugs used in medical practice. Decades of data support the safety of these agents, and with the availability of generic versions, costs of these medications are quite low. An initial study that encompassed analysis of cancer incidence in patients receiving statins found that it may increase risk for cancer [1]. Studies that followed showed that statins were not associated with increased cancer risk, and in fact may reduce the risk of several cancers, including breast cancer [2]. This finding that statins may reduce incidence of breast cancer is aligned with the corollary observation that the cholesterol synthesis pathway has been shown to be activated in the breast epithelium of women with high-risk breast lesions [3], and targeting the cholesterol pathway with statins inhibited breast cancer growth in mouse models [4–7]. Subsequent observational studies of the association between statin use and breast cancer incidence have yielded mixed results [2, 8, 9] and have not always accounted for confounding variables. A few pilot studies have tested statin efficacy in breast cancer, but these have offered limited insight due to use of non-informative biomarker endpoints or testing in a short preoperative window [10–14]. Thus, there is a need for high-quality preclinical data that address the potential for repurposing statins for breast cancer prevention and can inform subsequent clinical trial design.

In our previous work with fluvastatin, we studied its growth-inhibitory effects in vitro and in vivo using an MCF10A-based breast cancer progression model [15]. This model recapitulates many steps of human breast cancer progression such as atypia (MCF10.AT1 cells), ductal carcinoma in situ (DCIS) (MCF10.DCIS cells), and invasive breast cancer (MCF10.CA1D cells). In vitro experiments suggested fluvastatin to be preferentially more effective in impairing the growth of preneoplastic MCF10.AT1 compared to further progressed MCF10.DCIS cells. In MCF10.AT1-driven mouse xenograft experiments, we found fluvastatin inhibited tumor growth. Our efforts to understand the growth-inhibitory effects of fluvastatin in both in vitro and in vivo models revealed that statin treatment leads to feedback activation of the cholesterol pathway through upregulation of HMGCR protein and mRNA levels. These observations led us to hypothesize that more effective inhibition of the cholesterol pathway could be achieved though addition of another low-toxicity drug: aspirin, an activator of AMPK and a brake in the cholesterol pathway. We tested this dual targeting approach in vitro using MCF10.DCIS cells and found the fluvastatin and aspirin combination to effectively inhibit cell growth [15].

To further test this hypothesis in vivo, we studied the growth-inhibitory effect of fluvastatin alone, aspirin alone, or both in combination in SV40 C3TA_a_g, a transgenic mouse model of triple-negative breast cancer (TNBC). We report that although fluvastatin was confirmed as an effective strategy for breast cancer prevention, aspirin, alone or in combination with fluvastatin, did not show efficacy in this transgenic mouse model.

## Methods

### Cell line and treatments

Sub-confluent (175,000 cells/well) preneoplastic mammary epithelial cells (MCF10.NeoT and MCF10.AT1) and MCF10.DCIS were plated on coverslips in 6-well dishes and treated with 10 μM fluvastatin with or without the isoprenoid geranyl geranyl pyrophosphate (GGPP) (10 μM), mevalonic acid (100 μM), or squalene (50 μM) for 48 h. Total cellular proteins were extracted by lysing cells in RIPA buffer, described before [16].

### Animals

Four-week-old SV40C3 TAg hemizygous female mice (strain 013591, FVB-Tg(C3-1-TAg) cJeg/JegJ) were bought from The Jackson Laboratory. Mouse genotype was confirmed by qPCR-based testing for SV40T antigen. In hemizygous mice, the SV40 transgene is activated at about 4 weeks of age, causing p53 mutations and inactivation of the RB pathway. These changes lead to the development of atypia (starting at ~ 8 weeks), DCIS (by ~ 12 weeks), and eventually invasive breast cancer (as early as 16 weeks) [17]. After 1–2 weeks of quarantine and acclimatization, 5–6 week old mice, of approximately equal weight, were randomized into 4 groups. All experimental procedures were performed per guidelines of the institutional animal care and use committee.

### Mouse treatments and tumor measurements

In vivo studies were conducted in 2 batches of 5 mice/gp in each batch and 4 groups. Each of the four groups with a total of 10 mice/group were treated with (i) fluvastatin alone (10 mg/kg/day in water, which is equivalent to 48.6 mg (for an average adult weighing 60 Kg) and is well within the 20–80 mg/day range of fluvastatin to humans or (ii) aspirin alone (20 mg/kg/day dissolved in 200 μl of ethanol that was resuspended in 200 ml of water, final concentration of 0.001% ethanol), which is equivalent to 97.3 mg in adult humans, and is within the prescribed dose of 81–325 mg/day in humans or (iii) fluvastatin (10 mg/kg/day) + aspirin (20 mg/kg/day)-also referred as combo group, or (iv) vehicle control (0.001% ethanol). Statin and aspirin treatments were administered to mice in their drinking water, which was changed every other day. Water consumption/cage/day was logged, and the drug concentration adjusted accordingly to achieve the intended doses. Since the goal of this study was to investigate the chemopreventive effects of statin, aspirin, or both on breast cancer development, the drug treatments were started at an age 5–6 weeks of age before the onset of any visible lesions of breast cancer and continued for 16 weeks. Mice were weighed once a week and palpated twice a week for mammary tumors beginning at age 10 weeks until 21 weeks. Tumor/lesion size were measured using Vernier calipers. Any palpable mass of 3 mm or larger was considered a tumor and marked the onset of tumor in treatment groups. Tumor incidence at the time of necropsy was defined as the number of animals with macroscopic mammary lesions upon dissection at 22 weeks of age. While tumor burden was measured by tumor weight and the number of tumors/mice also referred as multiplicity of tumors/animal.

### Tissue processing

At the end of the 16-week treatment (age ~ 22 weeks), mice were euthanized regardless of tumor burden, and the mammary glands were harvested and weighed. From each mouse, 6 mammary glands (cervical, abdominal, and inguinal sites on the right and left sides of the body) were harvested and processed separately. Both tumor containing and tumor free glands were harvested. Each mammary gland was bisected, with one half fixed in formalin and embedded in paraffin (FFPE) and the second half snap-frozen in liquid nitrogen. FFPE blocks were cut into 5-μm-thick sections. From each block, the first and fifth sections were used for hematoxylin/eosin staining for histological confirmation of palpable tumors, and the second and third sections were used for the TUNEL assay and immunohistochemistry, respectively. RNA was extracted from snap-frozen samples for qPCR analyses.

### TUNEL assay and immunofluorescence

Apoptosis was measured at the single-cell level in the paraffin-embedded tissue sections or in single-cell suspension using the In Situ Cell Death Detection Kit (Sigma-Aldrich) labeled with TMR red fluorescence following the manufacturer’s instructions.

The tissue sections were first deparaffinized and serially rehydrated, permeabilized at room temperature, and incubated with TUNEL mixture at 37 °C for 1 h in the dark. Nuclei were stained with DAPI and sections were mounted using ProLong Diamond Antifade Mountant (ThermoFisher). TUNEL staining was analyzed using fluorescence microscopy. Eight slides (each containing at least 1 gland) per treatment group were stained. For each gland, multiple high-power (40×) images were taken. Alexa 488-labeled TUNEL-stained cells were overlaid with DAPI-stained nuclei (representing all cells). The average number of cells undergoing apoptosis was determined by counting the number of TUNEL-positive cells in a high-power field and normalizing with the total number of DAPI-positive cells.

### Annexin V and propidium iodide (PI) and fluorescence-activated cell sorting (FACS)

Apoptosis was measured in the vehicle and drug treated cells by staining cells with annexin V and PI using a Thermo Fisher kit following the manufacturer’s protocol. This assay is based on externalization of phosphatidyl serine (PS) on the plasma membrane, a hallmark of an apoptotic cell. Annexin V has a strong binding affinity towards PS and annexin V antibody is employed to detect cells that are undergoing apoptosis. Propidium iodide that is impermeant to cells is used in combination with annexin V to distinguish between apoptotic and necrotic cells. Dead/necrotic cells lose their membrane permeability and thus are positive for both annexin V and PI. MCF10.AT1 cells were treated with the various drugs for 2 days, then trypsinized, and the cell pellet was washed with PBS. Next, cells were incubated with Alexa Fluor™ 488 dye–labeled anti Annexin V antibody solution followed by staining with propidium iodide. Apoptotic cell fraction was determined by calculating annexin V -Alexa 488–labeled cells as a proportion of total live cells (unstained cells).

### Total cellular RNA extraction and qPCR

For total RNA extraction, snap-frozen mammary tissues were transferred to 2-ml tubes containing ceramic beads (OMNI International) in the presence of 0.5 ml Trizol (Bio-Rad) and were homogenized using an Omni Bead Ruptor 24 (OMNI International). The samples were lysed by running 2 cycles at the speed of 5.65 m/sec for 45 s, with a 30-s interval between each cycle. The tissue homogenates were spun at 10,000 rpm for 5 min at 4 °C, and the supernatant was processed using a Trizol (Bio-Rad) extraction method to extract RNA. To study mRNA levels, first-strand cDNA was synthesized using an iScript kit (Bio-Rad) as described previously [15]. mRNA expression of HMGCR, AMPK, and reference control -ribosomal protein L19 was measured by SYBR green-based chemistry (Bio-Rad) using the delta Ct (ΔΔCt) method as described before [15, 18, 19]. The expression levels of the genes of interest were measured by normalizing their signal (Ct) relative to loading control L19 and calculating the fold change in treated samples compared to vehicle control as the reference. The primer sequences for detecting mouse transcripts are provided in Supplementary Table 1.

### Immunohistochemistry

The levels of activated pAMPK were measured in the formalin fixed paraffin-embedded mammary tissue slides from the vehicle and fluvastatin + aspirin combination-treated mice by performing immunohistochemistry assay. Briefly, the slides were deparaffinized, antigen retrieval was performed and the pAMPK antibody was incubated at 1:150 dilution for 2 h followed by incubation with a secondary antibody labeled with DAB. Hematoxylin staining was performed to stain the nuclei. The cytoplasmic expression of pAMPK, which is known to be inversely correlated with histological grade and poor prognosis [20], was measured in the mammary glands by using Aperio imagescope cytoplasmic algorithm. The algorithm was fine-tuned with the help of pathologists to correctly measure the tissue staining. The staining score was evaluated and is presented as a scale from 0 to 3+, where 0 indicates no staining and 3+ strongest staining in the areas of interest that included mammary ducts. Ten slides per treatment group were stained and scored for pAMPK expression that is expressed as % cells stained/μm^2^.

### Cholesterol measurement

#### Long-term treatment

Total cholesterol levels in mouse blood were measured at the end of 16-week fluvastatin, aspirin, combination, and vehicle treatment. About 0.5 ml blood was drawn from mice by cardiac puncture (at the end of treatment as a terminal procedure) and allowed to sit at room temperature for about 2 h. Next, blood was centrifuged at about 1200 rpm for 20 min at 4 °C. Serum supernatant was collected and used for cholesterol measurements. Total cholesterol in serum was measured by using the Cholesterol/Cholesteryl Ester Assay Kit – Detection (Abcam) following the manufacturer’s protocol.

#### Short-term treatment

Five mice (about 10–11 weeks old) per group were treated with either fluvastatin alone (10 mg/kg/day) or vehicle control for 10 days in order to evaluate the effect of cholesterol pathway inhibition by fluvastatin by measuring total cholesterol levels in blood and mammary tissues. Blood samples were collected via retro-orbital bleeding prior to the start of treatment and 10 days after treatment, followed by serum extraction as detailed above. Mammary tissues were collected at the end of the 10-day study and bisected. One part was processed for RNA extraction, and the other part was weighed and snap-frozen for cholesterol measurement. Total cholesterol in mammary tissue homogenates and mouse serum was measured by using the Cholesterol/Cholesteryl Ester Assay Kit as described in the previous section.

### Western blotting

Total cellular proteins were subjected to SDS-PAGE gel electrophoresis as described previously [16]. The proteins were transferred to nitrocellulose membrane, which was then probed with PARP antibody that detects both total PARP (116 KDA) and cleaved PARP (89 KDa). The levels of cleaved PARP protein were quantified using odyssey imaging system and normalized with β-actin to determine the relative expression between vehicle and treated cells.

### Statistical analyses

Chi square and Fisher exact tests were applied to the tumor incidence data. The Wilcoxon rank sum test was used to compare the distributions of blood cholesterol levels between vehicle and fluvastatin treatment groups. The rest of the statistical analyses were performed using Student’s unpaired *t* test. All tests were two-sided. *p* values equal to or less than 0.05 were considered statistically significant.

## Results

### Fluvastatin effectively inhibits tumor incidence and growth in a TNBC mouse model, but aspirin does not add benefit

**Fig. 1:**
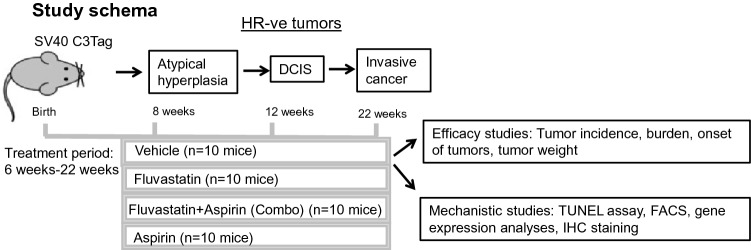
Schematic showing the schedule of drug treatments in SV40C3 TAg mouse model of breast cancer progression and the end point analyses. *HR* hormone receptor

**Fig. 2:**
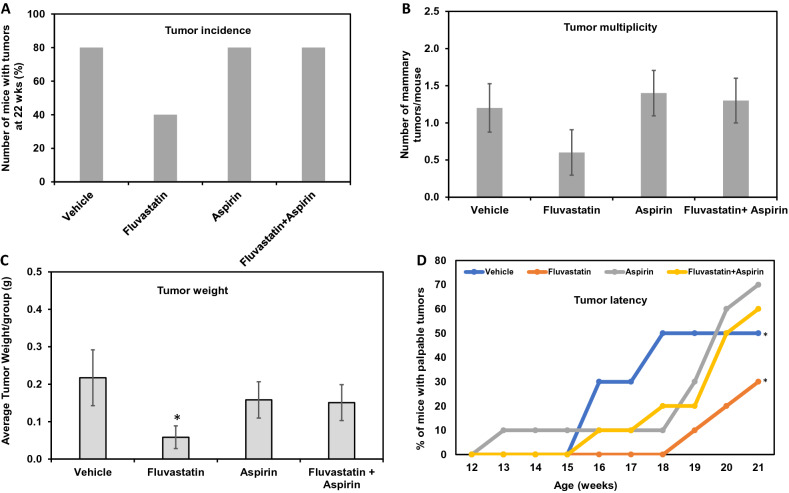
Fluvastatin treatment reduces tumor incidence, delays onset of tumor, and inhibits tumor growth in the SV40C3 TAg mouse model of breast cancer progression. **a **Fluvastatin treatment (10 mg/kg/day) for 16 weeks inhibited the percentage of mice that developed mammary tumors as determined by the macroscopic lesions at the time of necropsy at 22 weeks of age **b** and the average number of tumors per mouse relative to the vehicle control group after 16 weeks of treatment. **c **Fluvastatin treatment (10 mg/kg/day) for 16 weeks inhibited the average tumor weight per group **d **and delayed the average age at which tumors appear, as shown here as the % incidence of palpable tumor bearing mice during the course of study (12 week of age to 21 week age), relative to the vehicle control group. *p < 0.05

To investigate the effect of fluvastatin treatment alone or in combination with aspirin on the development of mammary tumors, SV40C3 TAg mice were treated with these agents for 16 weeks, and tumor incidence, tumor burden (as measured by weight and multiplicity of tumors/animal), and tumor latency were assessed between treatment groups (Fig. 1). Overall net change in body weight during16 weeks of treatment did not differ significantly between treatment groups (Supplementary Fig. 1). Fluvastatin treatment for 16 weeks (6 week–22 weeks) caused a 50% reduction in tumor incidence to 40%, as compared to 80% in all other treatment groups as revealed by macroscopic tumors present at the time of necropsy (all, *p* = 0.06) (Fig. 2a). In contrast to our in vitro data that suggested synergy [15] between the aspirin and fluvastatin combination, we observed no reduction in tumor incidence in mice treated with the combination of the 2 agents.

In addition to a lower tumor incidence, fluvastatin treatment resulted in a 50% reduction in the tumor burden as measured by multiplicity of tumors (0.6 tumors/mouse vs 1.2 tumors/mouse in vehicle-treated animals, Fig. 2b), although this difference between vehicle and fluvastatin-treated mice did not reach statistical significance (*p* = 0.2). Similar to the incidence data, the average tumor burden per mouse was comparable between the aspirin-treated (1.4 tumors/mouse), fluvastatin + aspirin–treated (1.3 tumors/mouse), and vehicle-treated (1.2 tumors/mouse) groups (Fig. 2b).

In mice that formed tumors, tumor burden as measured by weight was significantly reduced in the fluvastatin-treated animals relative to the vehicle-treated group (0.05 g vs 0.217 g, respectively; *p* = 0.04; Fig. 2c). In contrast, there was a non-significant 28–30% reduction in tumor weight in aspirin- and combination-treated groups relative to vehicle-treated animals (Fig. 2c).

Lastly, tumor latency was noted to be prolonged in fluvastatin- and combination-treated SV40C3 TAg mice, as suggested by tumor growth curves (Fig. 2d). The average times to spontaneous development of palpable mammary tumors were 16.8 weeks for controls, 20 weeks for fluvastatin treatment (*p* = 0.01 relative to vehicle), 18.75 weeks for aspirin treatment (*p* = non-significant), and 19.2 weeks for combination treatment (*p* = 0.02 relative to vehicle (Fig. 2d). Collectively, these results suggest that fluvastatin as a single agent is most effective at prevention, increasing the latency of tumor development and, importantly, reducing the frequency of tumor development. Although a combination of fluvastatin and aspirin also delayed the onset of tumors, the addition of aspirin to fluvastatin did not further abrogate tumor formation as compared to fluvastatin alone and in fact, provided no benefit in preventing tumors when compared to vehicle treatment.

### Pathway regulation by drugs

#### Cholesterol pathway inhibition by fluvastatin

**Fig. 3:**
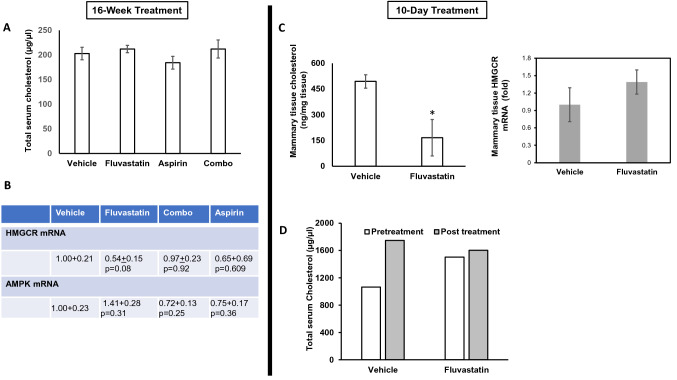
Fluvastatin treatment reduces total cholesterol in mouse mammary glands of SV40C3 TAg mice. **a** Bar diagram showing total serum cholesterol levels that did not change after 16-week treatment with fluvastatin (10 mg/kg/day), aspirin (20 mg/kg/day), or combined treatment relative to vehicle control. **b, c** Table and bar diagram (right side) showing HMGCR and AMPK mRNA levels in mammary tissue that did not change after 16-week or 10-day treatment with indicated drugs relative to vehicle control. The mean values were calculated by using the ΔΔCt method (as described previously [15]) after normalizing with ribosomal protein L19, which was set as 1. **d, c** Bar diagram showing total cholesterol levels before (in mouse serum) and after (in mouse serum and mammary tissue) short-term treatment with fluvastatin (10 mg/kg/day) relative to vehicle control. Values represent mean absolute cholesterol levels ± SEM. *p < 0.05

To verify the expected inhibition of the target pathway with these agents, we studied the extent of cholesterol pathway inhibition in the local (mammary tissue) and systemic (serum) environments of treated mice using a number of approaches. First, we examined the cholesterol levels in serum after 16 weeks of treatment and found no significant changes across the 4 treatment groups (Fig. 3a). These data were expected, in line with other studies that have demonstrated that a statin induced restorative feedback that triggers increased production of cholesterol elsewhere (e.g., hepatic), leading to an overall lack of change in serum cholesterol levels [21].

To determine the efficacy of statin inhibition of the cholesterol pathway at the local tissue level, we next examined the HMGCR mRNA levels in mouse mammary glands across all treatment groups. These analyses suggested no significant change in HMGCR mRNA levels in any of the treatment groups as compared to vehicle-treated group (Fig. 3b). This finding is in line with the known upregulation of this pathway through a restorative feedback response upon statin inhibition and suggests that HMGCR mRNA level is not an accurate measure of cholesterol pathway inhibition in treated animals.

We next sought to determine the ability of fluvastatin to target the cholesterol pathway by directly measuring cholesterol, a byproduct of the pathway, and to confirm that the lack of inhibition of HMGCR transcript levels in the mammary tissues after 16 weeks of treatment is due to compensatory feedback mechanisms over long-term treatment. We therefore tested for pathway inhibition after a short (10-day) course of treatment and measured tissue HMGCR RNA levels, tissue cholesterol levels as well as serum cholesterol levels. After treating mice with fluvastatin or vehicle control for 10 days, we found that fluvastatin lowered the average tissue cholesterol levels by ~ threefold relative to the vehicle-treated group (166 ng/mg tissue vs. 494 ng/mg, respectively; *p* = 0.03; Fig. 3c). This short-term study also revealed no change in HMGCR mRNA levels in the fluvastatin-treated group compared to the vehicle control group (Fig. 3c, right hand side) and confirmed that HMGCR transcript levels do not reflect cholesterol pathway inhibition. As in the 16-week study, the serum cholesterol levels did not change after 10-day fluvastatin treatment (Fig. 3d). Together, these results suggest that tissue cholesterol levels are a true measure of cholesterol pathway inhibition and fluvastatin does inhibit the cholesterol pathway in mammary tissue.

#### AMPK pathway regulation by aspirin

AMPK activation acts as a brake in the cholesterol pathway through direct inhibition of HMGCR and inhibition of the cholesterol pathway feedback response that is mediated through SREBP. To achieve better inhibition of the cholesterol pathway, we combined fluvastatin with aspirin, a known activator of AMPK signaling through increased AMPK phosphorylation [22]. In addition to the reported post-translational activation of AMPK, we found aspirin to upregulate AMPK transcript levels in vitro (Supplementary Table 2). To study AMPK pathway activation by 16-week treatment with aspirin alone or in combination with fluvastatin, we studied AMPK transcript levels in the mice mammary tissues by qPCR. Aspirin alone or combined with fluvastatin failed to significantly activate AMPK transcript levels (Fig. 3b) in the SV40C3 TAg mice. In order to confirm these results and the lack of efficacy of the fluvastatin + aspirin combination in tumor growth inhibition in vivo, we directly measured the pAMPK levels in mammary glands of mice treated with combination treatment relative to vehicle control mice. These immunohistochemical analyses showed the cytoplasmic pAMPK staining to be expressed in 73.24% mammary cells in fluvastatin + aspirin combination-treated mice compared to 78.8% positive cells in vehicle-treated mice tissues (Supplementary Fig. 2). Given the ubiquity of pAMPK in mammary cells, we explored whether inclusion of aspirin in the treatment regimen enriched for higher levels of pAMPK expression. However, we found no shift in the staining intensity gradient (0, 1+, 2+, 3+) in fluvastatin + aspirin-treated animals as compared to vehicle treatment, suggesting that the combination treatment failed to activate pAMPK (Supplementary Fig. 2) and thus explains its lack of efficacy.

### Fluvastatin inhibits mammary tumor growth through induction of apoptosis

**Fig. 4:**
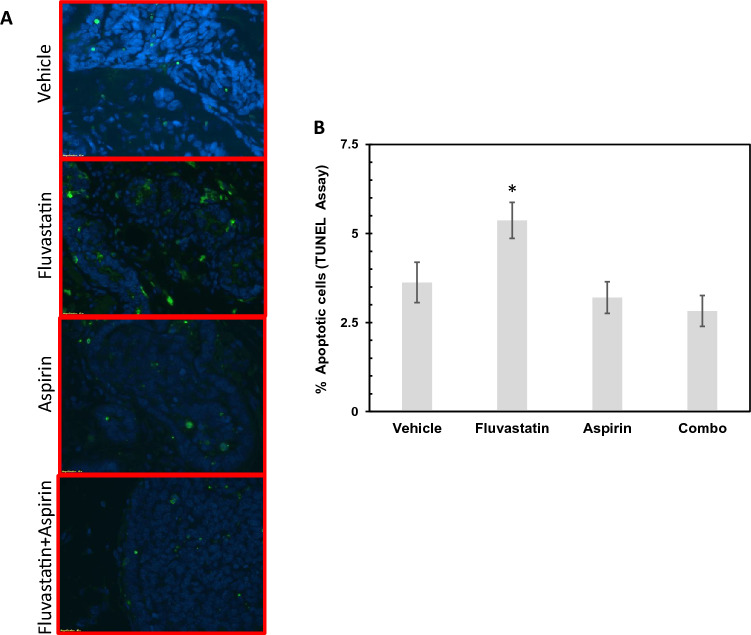
Tumor growth-inhibitory effects of fluvastatin are caused by increased apoptosis in mammary glands of a SV40C3 TAg mouse model of breast cancer progression. **a** Immunofluorescence images showing fluvastatin treatment (10 mg/kg/day) for 16 weeks that led to an increase in TUNEL-positive apoptotic cells (green); DAPI-stained nuclei (blue). **b** Bar diagram showing an increase in the proportion of apoptotic cells that are normalized to total number of DAPI-stained cells in the fluvastatin-treated group (10 mg/kg/day) relative to vehicle control group after 16 weeks of treatment. *p < 0.05

The growth-inhibitory properties of fluvastatin have been reported to be due to increased cell death in cell lines and mouse models [23–25]. To determine whether increased apoptotic cell death was indeed responsible for reduction in tumor growth in fluvastatin-treated mice, we performed TUNEL staining on a set of mammary glands (*n* = 8 samples per group) at the end of the 16-week treatment. Compared with the rate of TUNEL-positive apoptotic cells in vehicle control mice (3.62%), we found a significant increase in apoptotic cells in fluvastatin-treated mice (5.36%; *p* = 0.04) but not in aspirin- or combination-treated mice (3.20% and 2.82%, respectively, *p* = not significant) (Fig. 4). These results suggest that not only did aspirin alone not increase apoptotic cell death but that in combination with fluvastatin, it abrogated the effects of fluvastatin. These cellular findings are in line with the tumor incidence, tumor burden, and tumor latency data noted across the treatment groups.

### Fluvastatin-mediated cell death is reversed by addition of GGPP

**Fig. 5:**
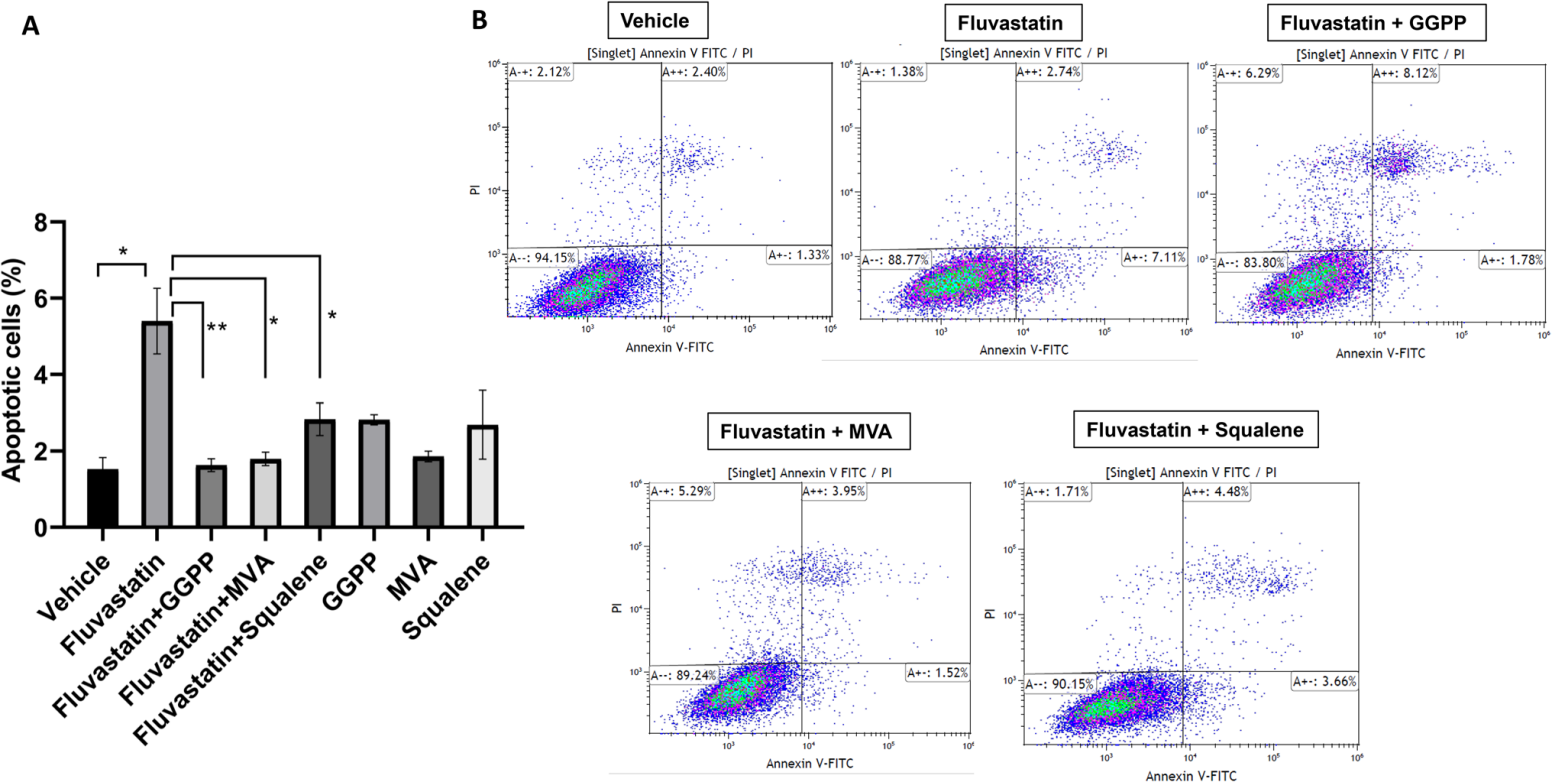
Replenishing with the cholesterol biosynthesis pathway metabolite, GGPP, inhibits the fluvastatin-induced apoptosis in MCF10.AT1 cells. **a** Bar diagram showing apoptotic cell death in fluvastatin (10 µM)-treated cells that were completely rescued by cotreatment with isoprenoid GGPP or MVA, but not squalene. **b** Raw FACS plots for treatment groups as shown. *p < 0.05; **p < 0.01

The cholesterol biosynthesis pathway leads to the production of cholesterol and non-sterol compounds such as GGPP (Supplementary Fig. 3). The homeostasis of GGPP and cholesterol is regulated differently, where cholesterol levels are sensed very precisely and remain unchanged due to multivalent feedback responses, whereas GGPP levels are more variable, depending on differential demand for intermediates of the cholesterol biosynthesis pathway [26–28]. Recent studies suggest that statins have cholesterol-independent effects [26–28]. We were therefore interested in identifying whether growth-inhibitory effects of statins are mediated through GGPP in our tumor model system. We hypothesized that fluvastatin-mediated induction of apoptotic cell death in tumor tissue is driven by depletion of GGPP and turned to a cell line-based model system to test this concept. MCF10.AT1 cells were treated for 48 h with fluvastatin alone or in combination with either GGPP; mevalonate (MVA), an upstream substrate; or squalene, a precursor of cholesterol downstream of GGPP and MVA. As shown in Fig. 5, we found fluvastatin to induce apoptosis (5.40% in fluvastatin vs 1.52% in vehicle control treated cells, *p* = 0.01) as measured by FACS assay. Replenishing GGPP to fluvastatin-treated cells blocked apoptotic cell death (5.40% in fluvastatin vs 1.63% in fluvastatin + GGPP, *p* < 0.01) (Fig. 5) and cells look as healthy as vehicle-treated cells (Supplementary Fig. 4). Supplementation with mevalonate and GGPP completely reversed fluvastatin-induced cell death to levels seen in vehicle control (1.63%, 1.79% for GGPP and MVA, respectively, *p* < 0.05 compared to 5.40% in cells treated with fluvastatin alone). Partial reversal of fluvastatin-induced apoptosis was seen with the addition of squalene (2.83%).

**Fig. 6:**
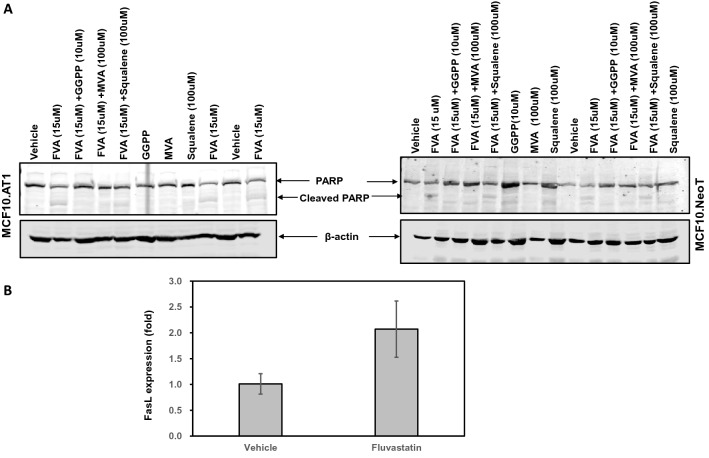
Apoptotic cell death caused by fluvastatin is mediated via FAS-FASL pathway. **a** Western blotting images showing an upregulation in cleaved PARP by fluvastatin treatment to MCF10.AT1 and MCF10.NeoT cells that were completely rescued by GGPP and MVA, but not squalene. **b** Bar diagram showing an increase in the FASL transcript levels that are normalized to ribosomal protein L19 (RPL19) in the fluvastatin-treated group (10 mg/kg/day) relative to vehicle control group after 16 weeks of treatment. Values represent mean ± SEM, *p* = 0.06

In order to confirm if the apoptotic cell death caused by fluvastatin is characterized by PARP cleavage- a hallmark of apoptosis- we performed western blotting and measured PARP cleavage by fluvastatin treatment in MCF10.NeoT and MCF10.AT1 cells. We found fluvastatin treatment to cause PARP cleavage (10.57 fold in MCF10.NeoT and 7.0 -fold in MCF10.AT1 cells), and GGPP and MVA to completely inhibit the PARP cleavage (Fig. 6a, Supplementary Fig. 5). Consistent with FACS results, western blotting also showed the squalene to partially reverse the apoptosis caused by fluvastatin as indicated a 25.20% and 59.30% reduction in cleaved PARP in MCF10.NeoT and MCF10.AT1 cells, respectively (Fig. 6a, Supplementary Fig. 5).

These findings support the idea that fluvastatin-induced reduction of GGPP is a major cause of reduced apoptosis in statin treated transgenic mice and in turn may be the primary driver of the efficacy of statin chemoprevention.

### Fluvastatin-mediated induction in apoptosis is caused via an increase in FASL expression

The interaction and activation of FAS-FASL is an important step that is responsible for initiating apoptosis. FAS-FASL pathway activation is finely regulated and transcriptional upregulation of FASL is one of the key regulatory mechanisms [29]. Statin inhibition of RhoA GTPAse, that is prenylated through GGPP, is known to activate FASL expression [30] (Supplementary Fig. 3). Therefore, we tested if fluvastatin-induced apoptosis is mediated via increase in FASL transcript expression and found a twofold induction (*p* = 0.06) in FASL expression in mammary tissues of the fluvastatin-treated mice relative to vehicle control tissue (Fig. 6b) suggesting a role of FAS-FASL pathway in fluvastatin-induced apoptosis.

## Discussion

In the present study, we found the cholesterol-lowering drug fluvastatin delayed the onset of mammary tumors, lowered tumor incidence, and decreased tumor burden in a transgenic mouse model of breast cancer through induction of apoptosis. In contrast, aspirin, had no efficacy in breast cancer prevention, and the combination of the statin and aspirin was no better than the statin alone. While the efficacy of fluvastatin is mediated by the expected targeting of HMGCR enzymatic activity, our data also suggest a role for GGPP inhibition as a potential downstream target as a mechanism for efficacy. These findings of the efficacy of a statin for breast cancer prevention further validate our prior preclinical work using in vitro models and xenograft studies.

Our findings are in line with a previous publication by Green and co-workers showing that lovastatin, another lipophilic statin, has efficacy in SV40C3 TAg mice [31]. However, Green et al. reported a reduction only in in situ lesions, not invasive carcinomas, with lovastatin treatment. It should be noted, however, that in the work reported by Green et al., the treatment regimen was shorter than our studies, with animals treated for only 4 weeks starting at 12 weeks of age [31]. Although invasive cancers are described at 16 weeks of age in this mouse model, the prevalence of invasive cancers at 16 weeks is overall low, and therefore precludes a definitive conclusion of efficacy of statin treatment on the endpoint of invasive tumors. More relevant to our findings are the survival outcomes that were reported by Green et al. with longer period (4 months) of lovastatin treatment, where more animals were alive in the lovastatin-treated group than in the control group [31]. These survival data suggest that lovastatin-treated animals had fewer invasive cancers after 4 months of treatment and are thus consistent with the lower incidence and lower burden of disease we note in our fluvastatin-treated animals at 5 months of age compared to control animals. Our report builds on these studies by providing mechanistic details to the tumor growth-inhibitory effects of fluvastatin in SV40C3TAg mice.

While the cholesterol biosynthesis pathway is well known for the production of cholesterol, which is required as a building block for the assembly of cell membranes of rapidly dividing cancer cells, lately focus has also shifted to recognizing this pathway as important for production of isoprenoids such as GGPP and farnesyl pyrophosphate (FPP) [26–28]. GGPP drives proper membrane localization, function, and activation of GTPases rac and rho [32] and is also involved in cell migration and micropinocytosis [28].

Overexpression of GGPP leads to aberrant activation of rac/rho–driven oncogenic ERK and/or AKT signaling, which causes increased cell proliferation and cell migration [33]. Consistently, drugs that cause depletion of GGPP decrease cell proliferation, inhibit cell migration, block micropinocytosis, starve cells and induce apoptotic cell death in model systems including organoids driven from SV40C3 TAg mice. [28]. Fluvastatin-induced apoptotic cell death that we observed in the present study could be due to either (i) direct inhibition of HMGCR enzymatic activity, which would lead to changes in both sterol and non-sterol (GGPP) downstream products of the pathway, or (ii) direct inhibition of GGPP. We found statin induced GGPP depletion to cause an upregulation of apoptosis that was evident by an increase in cleaved PARP in fluvastatin-treated MCF10.AT1 and MCF10.DCIS cells. Our MCF10.AT1 and MCF10.DCIS -based rescue experiments thus have clearly suggested that apoptotic effects are mediated at least in part through GGPP. While there are multiple pathways through which GGPP depletion can cause apoptotic cell death, we found an upregulation in FASL mediated apoptosis to play a role for growth-inhibitory effects of fluvastatin observed in our SV40C3 TAg mice experiments These results are consistent with the growing evidence that the beneficial effects of statins are beyond cholesterol modulation and partly mediated through isoprenoid inhibition in the setting of neurological disorders and cancers [26–28].

Aspirin is a safe and inexpensive drug that could be repurposed for prevention of cancer alone or in combination with other drugs. In the current study, we hypothesized that combining aspirin would potentiate the efficacy of fluvastatin, but we did not see any further benefit of aspirin addition; rather- it diminished the growth-inhibitory effects of fluvastatin. Our qPCR-based analyses of AMPK transcript and direct measurement of pAMPK levels in mouse mammary tissues of vehicle and combination-treated mice reveal no upregulation of pAMPK levels by long-term aspirin treatment and thus explains the lack of additive benefit of aspirin in the fluvastatin + aspirin combination group. However, it remains possible that a different route of aspirin administration, a different aspirin dose or another pAMPK activator might still be able to increase the chemopreventive efficacy of fluvastatin.

Lastly, some of the pleiotropic effects of statins are known to be mediated through other mechanisms that involve reducing oxidative stress, reducing inflammation, and modulating the immune environment [34–36]. The role of FAS-FASL pathway in maintaining a balance between cell survival and cell death as well as in immune homeostasis [29], along with our observed upregulation of FASL in fluvastatin-treated mice, suggest the potential involvement of immune environment in tumor growth inhibition seen with statin treatment that needs to be explored. Recently, statins have been reported to enhance the growth-inhibitory effects of dendritic cell-based Th1 cytokine therapy and suppress tumor growth in a mouse model of Her2+ breast cancer [36]. Complete understanding of these off-target effects of statins would allow their rationale use in combination with other existing drugs to achieve better efficacy for breast cancer prevention.

## Conclusions

The present study confirms the growth-inhibitory effects of fluvastatin in a mouse model of hormone-negative breast cancer and suggests its potential use as a chemopreventive agent in high-risk breast cancer patients.

## Supplementary Information

Below is the link to the electronic supplementary material.

Supplementary file1 (PPTX 12479 kb)

## Data Availability

Data sharing is not applicable to this article as no datasets were generated or analyzed during the current study.
