# Supplementary material for: Efficacy of fluvastatin and aspirin for prevention of hormonally insensitive breast cancer
Source: Breast Cancer Res Treat. 2021 Apr 24;187(2):363–74. doi: 10.1007/s10549-021-06229-0 (PMC8190001; doi:10.1007/s10549-021-06229-0)
Supplement: Supplementary file 1 — Supplementary file1 (PPTX 12479 kb) [file 10549_2021_6229_MOESM1_ESM.pptx]

## Slide 1
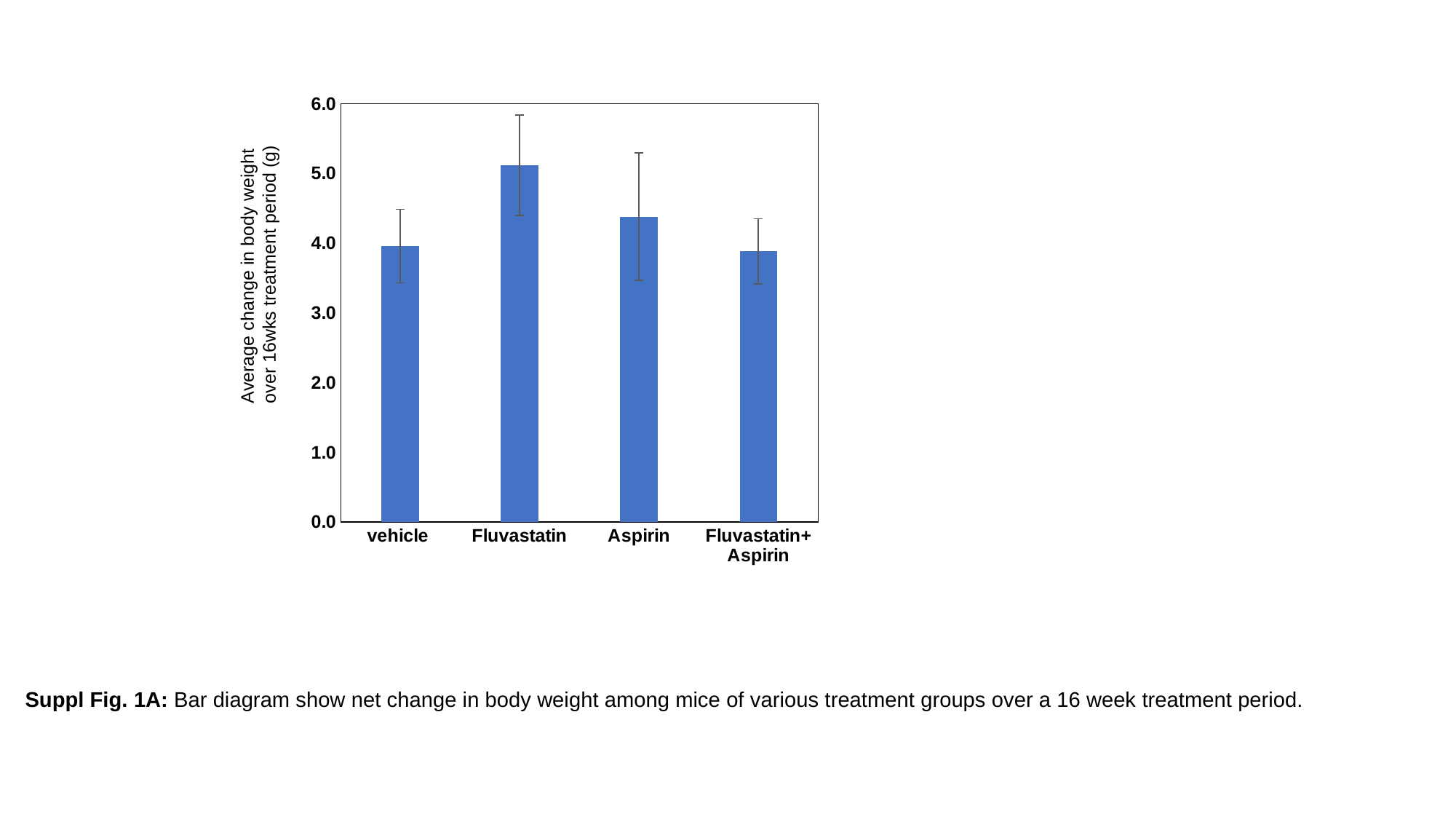

### Chart
| Category | Mean |
|---|---|
| vehicle | 3.96 |
| Fluvastatin | 5.118 |
| Aspirin | 4.383 |
| Fluvastatin+Aspirin | 3.8850000000000007 |Average change in body weight
over 16wks treatment period (g)
Suppl Fig. 1A: Bar diagram show net change in body weight among mice of various treatment groups over a 16 week treatment period.

## Slide 2
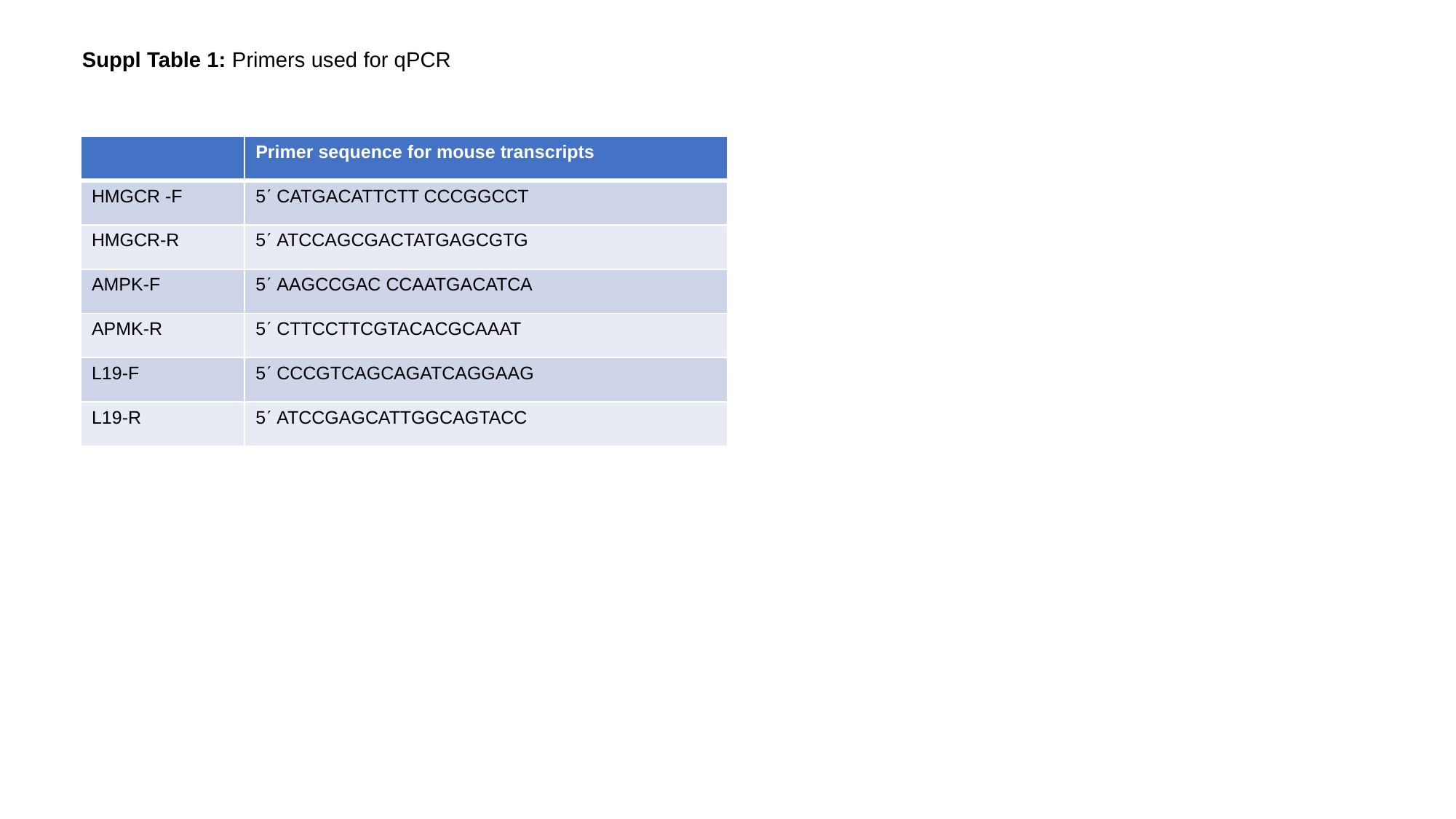

Suppl Table 1: Primers used for qPCR
| | Primer sequence for mouse transcripts |
| --- | --- |
| HMGCR -F | 5′ CATGACATTCTT CCCGGCCT |
| HMGCR-R | 5′ ATCCAGCGACTATGAGCGTG |
| AMPK-F | 5′ AAGCCGAC CCAATGACATCA |
| APMK-R | 5′ CTTCCTTCGTACACGCAAAT |
| L19-F | 5′ CCCGTCAGCAGATCAGGAAG |
| L19-R | 5′ ATCCGAGCATTGGCAGTACC |

## Slide 3
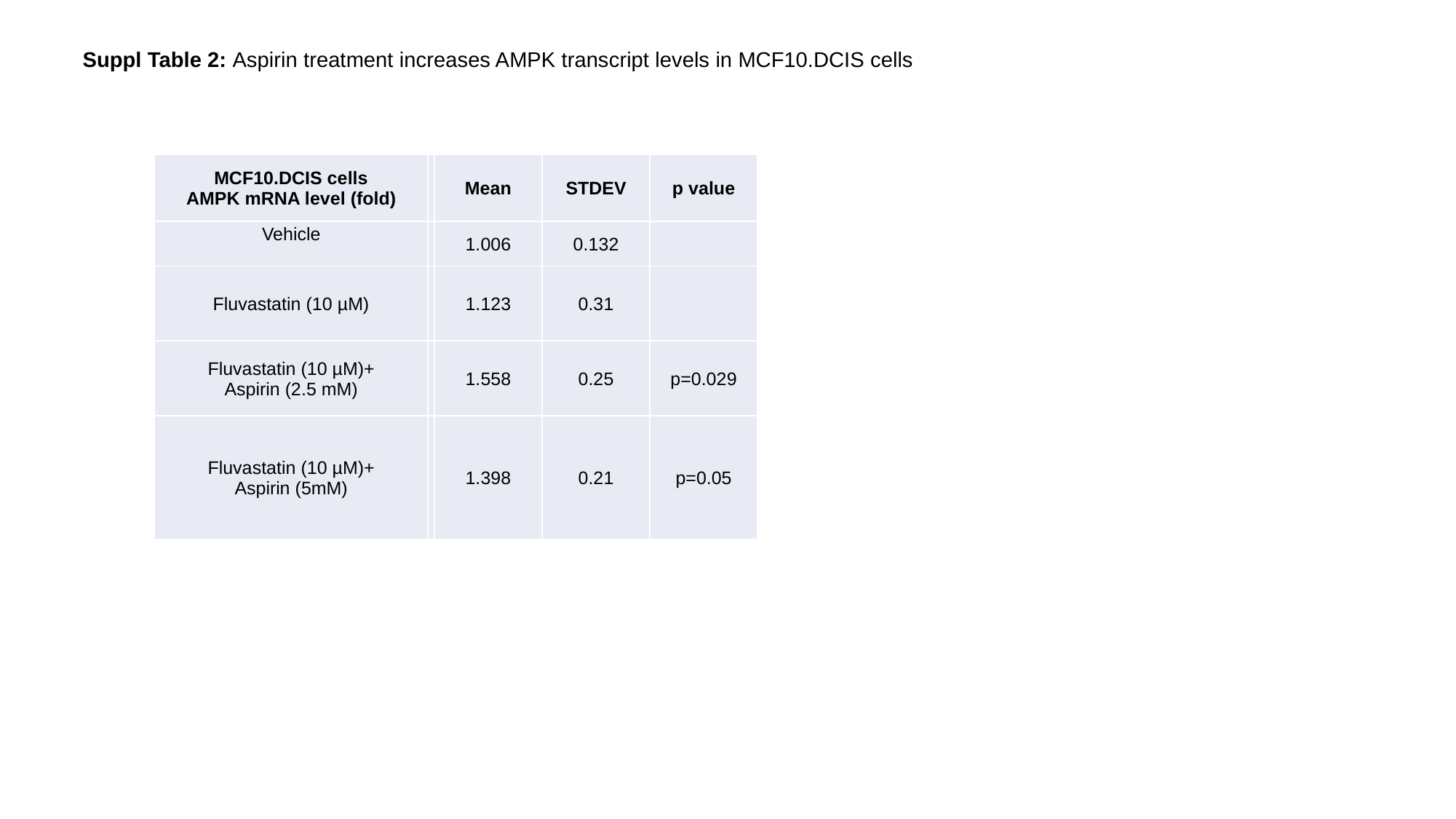

Suppl Table 2: Aspirin treatment increases AMPK transcript levels in MCF10.DCIS cells
| MCF10.DCIS cells AMPK mRNA level (fold) | | Mean | STDEV | p value |
| --- | --- | --- | --- | --- |
| Vehicle | | 1.006 | 0.132 | |
| Fluvastatin (10 µM) | | 1.123 | 0.31 | |
| Fluvastatin (10 µM)+ Aspirin (2.5 mM) | | 1.558 | 0.25 | p=0.029 |
| Fluvastatin (10 µM)+ Aspirin (5mM) | | 1.398 | 0.21 | p=0.05 |

## Slide 4
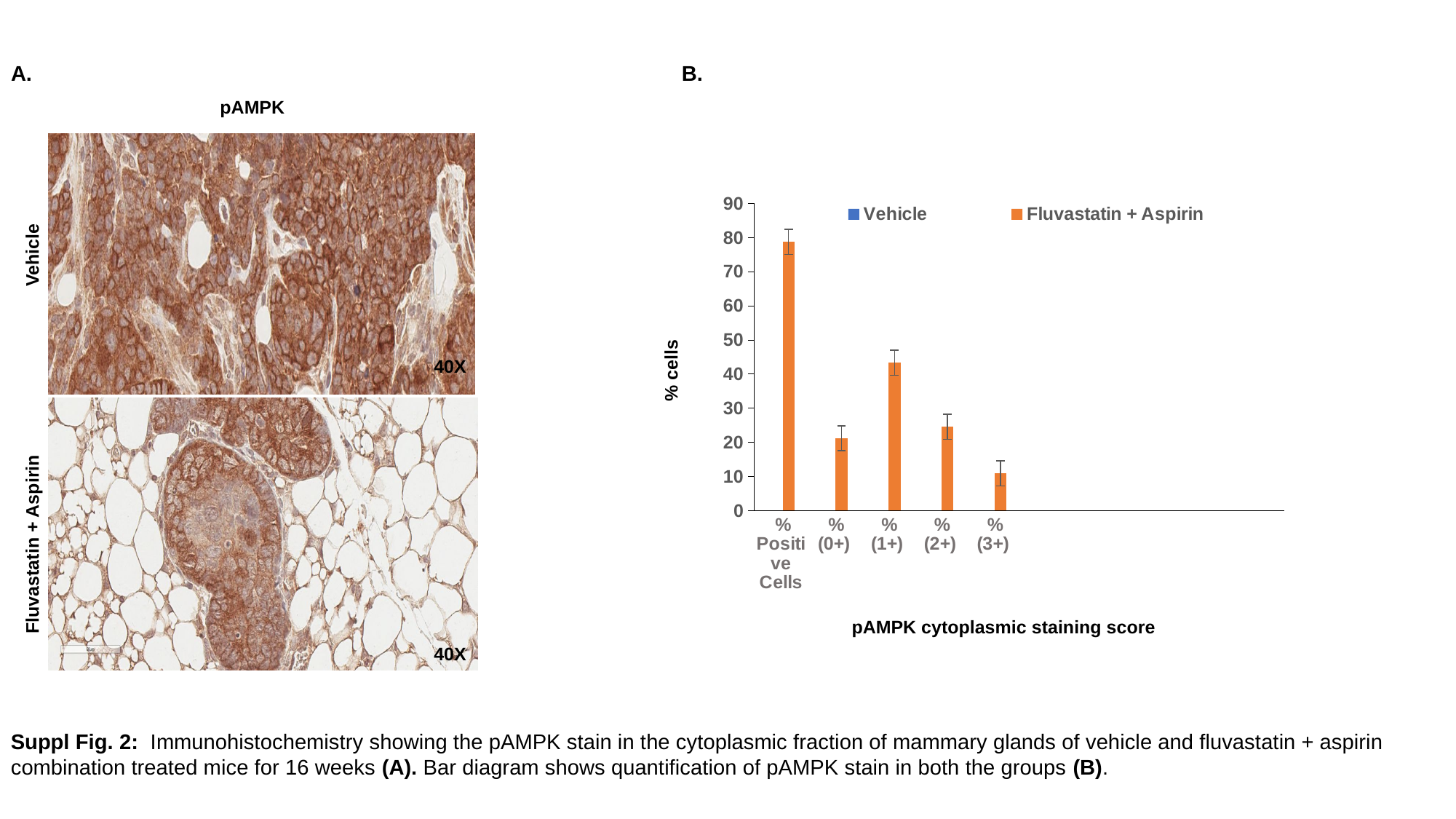

A.
B.
pAMPK
### Chart
| Category | Vehicle | Fluvastatin + Aspirin |
|---|---|---|
| % Positive Cells | 73.235675 | 78.7998 |
| % (0+) | 26.76432375 | 21.20019125 |
| % (1+) | 37.953725 | 43.3193625 |
| % (2+) | 28.238834999999998 | 24.5488125 |
| % (3+) | 7.04307925 | 10.931652875 |Vehicle
40X
% cells
Fluvastatin + Aspirin
pAMPK cytoplasmic staining score
40X
Suppl Fig. 2: Immunohistochemistry showing the pAMPK stain in the cytoplasmic fraction of mammary glands of vehicle and fluvastatin + aspirin combination treated mice for 16 weeks (A). Bar diagram shows quantification of pAMPK stain in both the groups (B).

## Slide 5
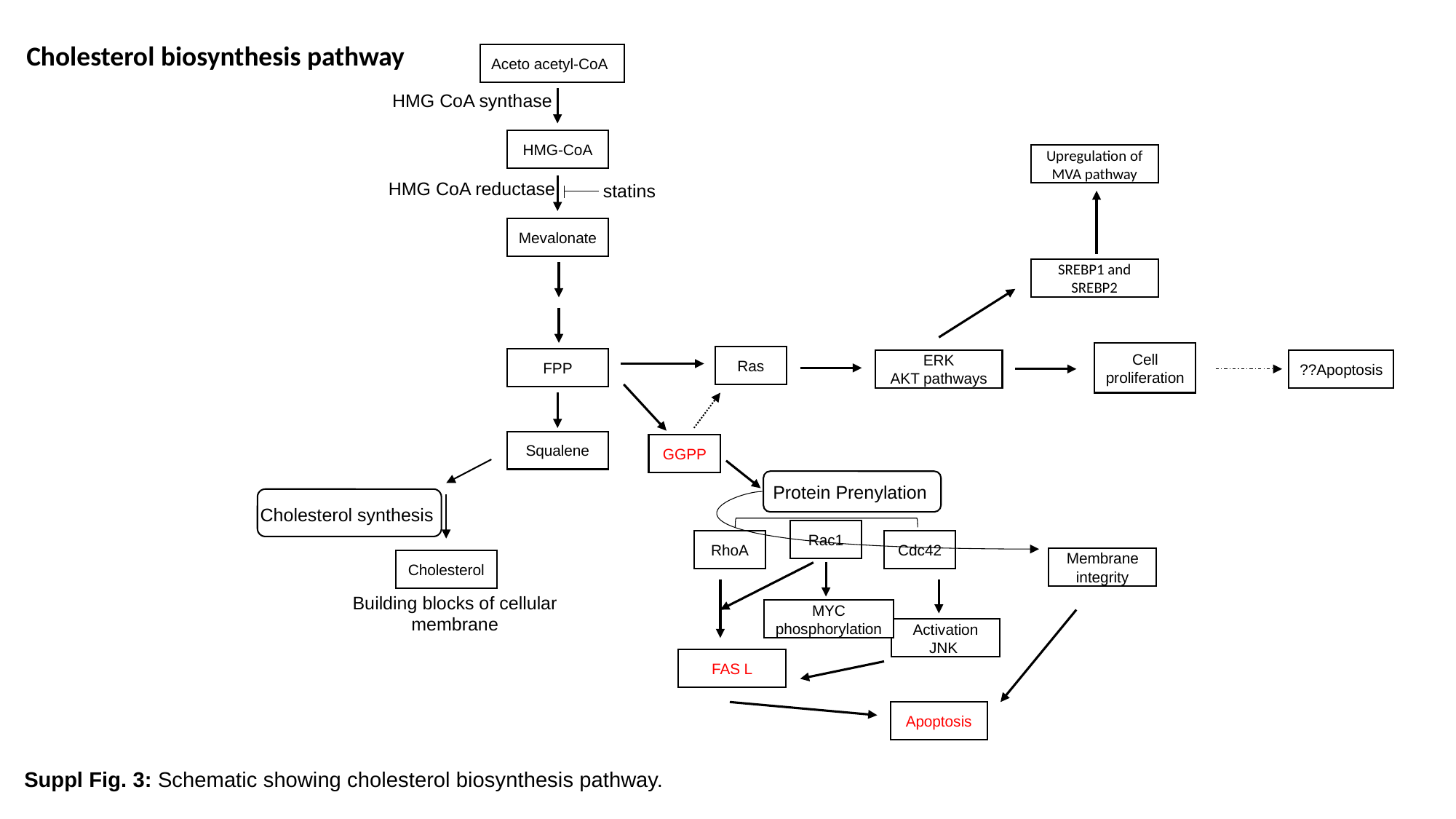

Cholesterol biosynthesis pathway
Aceto acetyl-CoA
HMG CoA synthase
HMG-CoA
Upregulation of MVA pathway
HMG CoA reductase
statins
Mevalonate
SREBP1 and SREBP2
Cell proliferation
Ras
FPP
ERK
AKT pathways
??Apoptosis
Squalene
GGPP
Protein Prenylation
Cholesterol synthesis
Rac1
RhoA
Cdc42
Membrane integrity
Cholesterol
Building blocks of cellular membrane
MYC phosphorylation
Activation JNK
FAS L
Apoptosis
Suppl Fig. 3: Schematic showing cholesterol biosynthesis pathway.

## Slide 6
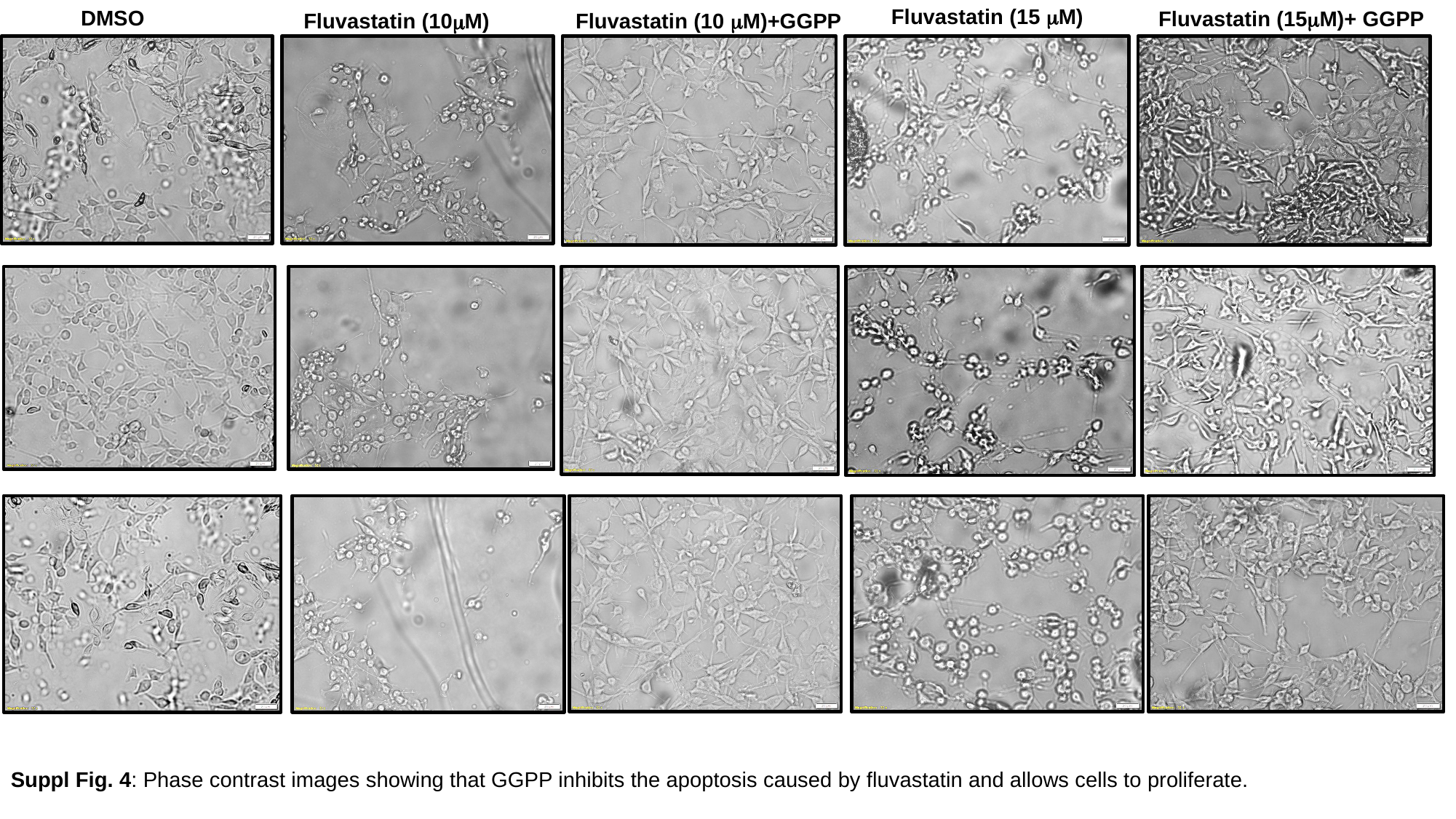

Fluvastatin (15μM)+ GGPP
Fluvastatin (15 μM)
DMSO
Fluvastatin (10 μM)+GGPP
Fluvastatin (10μM)
Suppl Fig. 4: Phase contrast images showing that GGPP inhibits the apoptosis caused by fluvastatin and allows cells to proliferate.

## Slide 7
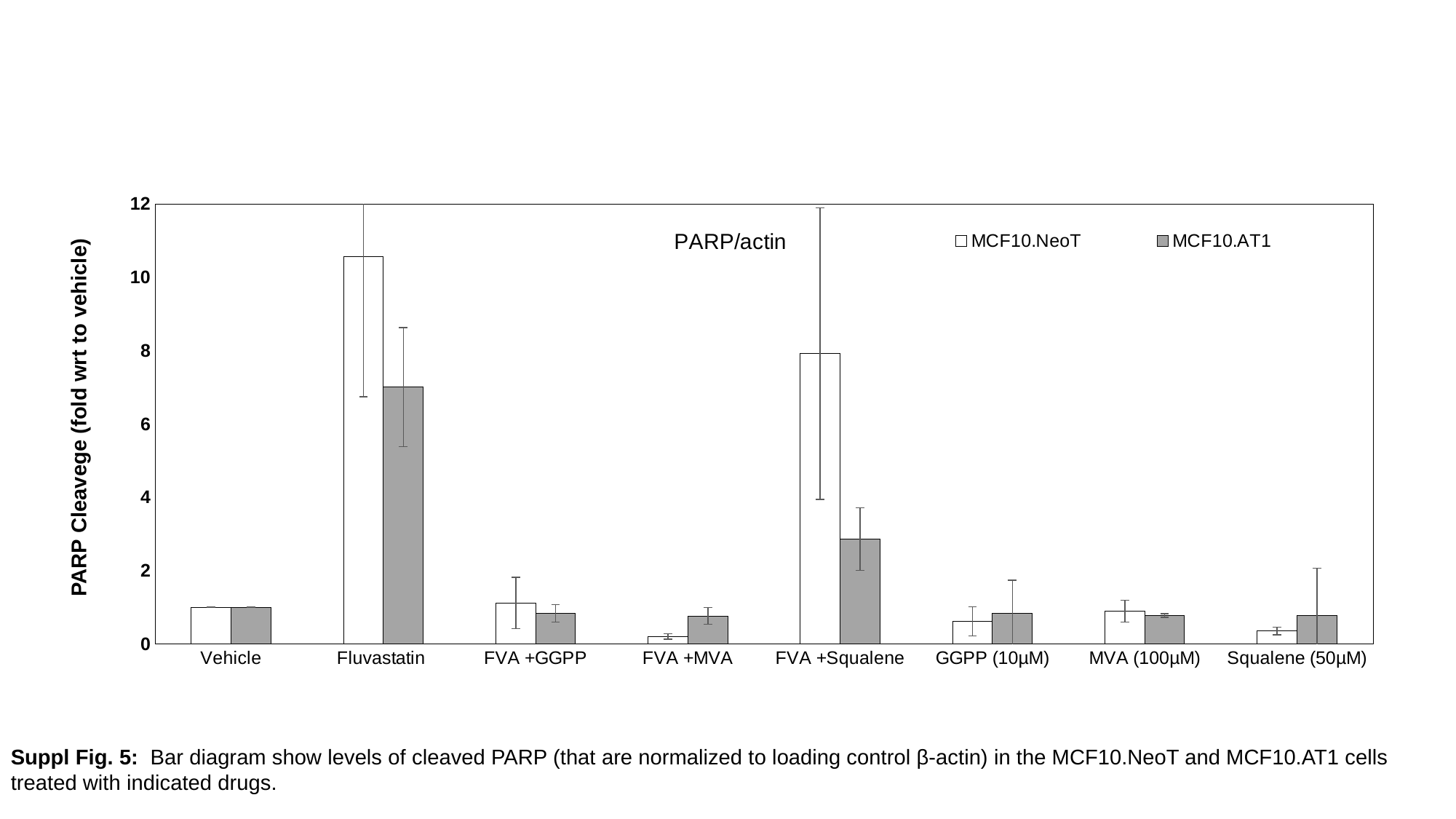

### Chart: PARP/actin
| Category | MCF10.NeoT | MCF10.AT1 |
|---|---|---|
| Vehicle | 1.0 | 1.0 |
| Fluvastatin | 10.571265957378925 | 7.005208963030509 |
| FVA +GGPP | 1.1168699605578032 | 0.83714235304647 |
| FVA +MVA | 0.20758386051720112 | 0.7617356304727645 |
| FVA +Squalene | 7.919749064231208 | 2.858826009195699 |
| GGPP (10µM) | 0.616788387342253 | 0.8437036528949958 |
| MVA (100µM) | 0.8973929003052528 | 0.7737380838342144 |
| Squalene (50µM) | 0.3539056271113248 | 0.7790861158379033 |PARP Cleavege (fold wrt to vehicle)
Suppl Fig. 5: Bar diagram show levels of cleaved PARP (that are normalized to loading control β-actin) in the MCF10.NeoT and MCF10.AT1 cells treated with indicated drugs.
